# *Plasmodium falciparum* AMA1 and CSP antigen diversity in parasite isolates from southern Ghana

**DOI:** 10.3389/fcimb.2024.1375249

**Published:** 2024-05-13

**Authors:** Kwadwo A. Kusi, Linda E. Amoah, Festus Kojo Acquah, Nana Aba Ennuson, Abena F. Frempong, Ebenezer A. Ofori, Kwadwo Akyea-Mensah, Eric Kyei-Baafour, Frank Osei, Augustina Frimpong, Susheel K. Singh, Michael Theisen, Edmond J. Remarque, Bart W. Faber, Maria Belmonte, Harini Ganeshan, Jun Huang, Eileen Villasante, Martha Sedegah

**Affiliations:** ^1^ Department of Immunology, Noguchi Memorial Institute for Medical Research, College of Health Sciences, University of Ghana, Legon, Ghana; ^2^ Center for Medical Parasitology at the Department of International Health, Immunology and Microbiology, University of Copenhagen, Copenhagen, Denmark; ^3^ Department of Congenital Diseases, Statens Serum Institut, Copenhagen, Denmark; ^4^ Department of Parasitology, Biomedical Primate Research Center, Rijswijk, Netherlands; ^5^ Henry M. Jackson Foundation for the Advancement of Military Medicine, Inc., Bethesda, MD, United States; ^6^ Malaria Department, Naval Medical Research Command, Silver Spring, MD, United States

**Keywords:** polymorphism, Ghana, plasmodium, malaria, PfAMA1, PFCSP

## Abstract

**Introduction:**

Diversity in malarial antigens is an immune evasion mechanism that gives malaria parasites an edge over the host. Immune responses against one variant of a polymorphic antigen are usually not fully effective against other variants due to altered epitopes. This study aimed to evaluate diversity in the Plasmodium falciparum antigens apical membrane antigen 1 (PfAMA1) and circumsporozoite protein (PfCSP) from circulating parasites in a malaria-endemic community in southern Ghana and to determine the effects of polymorphisms on antibody response specificity.

**Methods:**

The study involved 300 subjects, whose *P. falciparum* infection status was determined by microscopy and PCR. Diversity within the two antigens was evaluated by msp2 gene typing and molecular gene sequencing, while the host plasma levels of antibodies against PfAMA1, PfCSP, and two synthetic 24mer peptides from the conserved central repeat region of PfCSP, were measured by ELISA.

**Results:**

Of the 300 subjects, 171 (57%) had *P. falciparum* infection, with 165 of the 171 (96.5%) being positive for either or both of the *msp2* allelic families. Gene sequencing of DNA from 55 clonally infected samples identified a total of 56 non-synonymous single nucleotide polymorphisms (SNPs) for the *Pfama1* gene and these resulted in 44 polymorphic positions, including two novel positions (363 and 365). Sequencing of the Pfcsp gene from 69 clonal DNA samples identified 50 non-synonymous SNPs that resulted in 42 polymorphic positions, with half (21) of these polymorphic positions being novel. Of the measured antibodies, only anti-PfCSP antibodies varied considerably between PCR parasite-positive and parasite-negative persons.

**Discussion:**

These data confirm the presence of a considerable amount of unique, previously unreported amino acid changes, especially within PfCSP. Drivers for this diversity in the Pfcsp gene do not immediately seem apparent, as immune pressure will be expected to drive a similar level of diversity in the *Pfama1* gene.

## Introduction

Malaria remains a disease of public health importance as it still accounts for high levels of morbidity and mortality and it places a huge economic burden on endemic countries, most of which are classified as low-income countries (WHO, 2020). Significant gains have been made in disease control efforts over the last two decades through a concerted deployment of available control and prevention tools, but these gains seem to have plateaued in the last five years (WHO, 2020). Effective malaria vaccines will be an important addition to these control tools, especially with an increasing number of endemic countries entering the pre-elimination and elimination phases. Vaccine development, however, requires a good understanding of both parasite and host biology, and one important factor that has hindered the rapid development of highly effective vaccines is the existence of numerous parasite variants in many endemic communities. One major source of these differences amongst variants is point mutations in antigens, especially those that are essential for parasite survival and, hence, come under immune pressure (Ferreira et al., 2004; Farooq et al., 2009). These point mutations result in the existence of different polymorphic forms of the same antigens, a phenomenon that has been linked with the parasite’s strategy to evade immune responses directed at these antigens (Ferreira et al., 2004; Mahajan et al., 2005). Two such vaccine candidate antigens that show significant polymorphisms are *P. falciparum* apical membrane antigen 1 (AMA1) and circumsporozoite protein (CSP). *P. falciparum* AMA1 (PfAMA1) is an antigen expressed in the parasite micronemes but is exported to the parasite apical surface around the time of red cell invasion (Besteiro et al., 2011). The antigen is found on the liver and red blood cell (RBC) infective stages of the parasite and its interactions with the Rhoptry neck protein (RON) complex within the RBC membrane contributes to tight junction formation as part of the host cell invasion process (Lamarque et al., 2011; Srinivasan et al., 2011). Polymorphisms in PfAMA1 have been shown to significantly reduce the efficacy of PfAMA1-based vaccines (Remarque et al., 2012; Ouattara et al., 2013). The other antigen, PfCSP, is expressed on the sporozoite surface and is the most abundant antigen in that stage of the parasite. PfCSP plays a host liver cell adhesive function that promotes sporozoite infectivity to these cells following mosquito inoculation (Frevert et al., 1993; Coppi et al., 2007; Coppi et al., 2011). Polymorphisms in PfCSP have also been shown to affect PfCSP-based vaccine efficacy, with vaccine induced immune responses being less effective against parasites expressing genetically distant PfCSP variants (Neafsey et al., 2015; Pringle et al., 2018).

The capacity of these antigens as malaria vaccine targets is therefore greatly limited by the presence of different parasite variants within any endemic population. Limited heterologous protection has also been shown in some studies with sporozoite vaccines (Epstein et al., 2017; Walk et al., 2017). Thus, depending on the genetic distance between antigens or parasites used for vaccine formulation and those of parasites circulating in any geographical region, the quality of vaccine-induced immune responses and, by extension, the level of protection that such vaccines will offer, can be greatly compromised. An in-depth assessment of the impact of parasite diversity across different geographical regions will contribute to our understanding of the mechanisms underlying immune response induction in these complex situations. It will also greatly enhance our chances of rapidly developing cost-effective vaccines with parasite strain-transcending properties. There is, however, very limited data on diversity within important vaccine candidate antigens in Ghana and some other parts of sub-Saharan Africa, hence the need for the current analyses.

The aim of this study was therefore to assess the genetic diversity in two important malaria vaccine candidate antigens, PfCSP and PfAMA1, from circulating parasites in a malaria-endemic community in southern Ghana, and to determine the effects of the identified polymorphisms on antibody response specificity. We analyzed samples from participants with clonal parasite infections for diversity in the *Pfama1* and *Pfcsp* genes and, herein, report the existence of a huge diversity of parasites within a community with moderate to high malaria transmission.

## Methods

### Ethics

This study was approved by the Institutional Review Board at Noguchi Memorial Institute for Medical Research (NMIMR, protocol approval number 067/18-19). Research data is derived from an approved NMIMR Institutional Review Board protocol number 067/18-19. The Naval Medical Research Command’s (NMRC) involvement in the study was determined to be non-human subject research by the NMRC IRB. Both NMIMR and NMRC have a United States Government Federalwide Assurance from the Office for Human Research Protections. The protocol was conducted in accordance with the principles in The Belmont Report and federal regulations regarding the protection of human subjects in research including 32 CFR 219 (The Common Rule), and all regulations pertinent to the Department of Defense, the Department of the Navy, the Bureau of Medicine and Surgery of the United States Navy and internal NMRC policies for human subject protections and responsible conduct of research. All NMRC and NMIMR personnel contributing to or performing human research were certified as having completed human research ethics education and training. Written informed consent was sought from all study subjects who willingly agreed to be part of the study and met the inclusion criteria.

### Study area

The study was conducted in Obom in the Ga South District of the Greater Accra Region of Ghana. Obom is a peri-urban community that is approximately 25 Km from Accra, the capital of Ghana, and residents of this community are mostly peasant farmers. Malaria transmission in the area is seasonal, with the major transmission period being typically from May to October, followed by a period of limited transmission from November to April the following year. At the peak of transmission the average parasite prevalence within the community was estimated to be 34% in 2019, with most infected persons being asymptomatic (Abukari et al., 2019).

### Sample collection

This cross-sectional study was conducted between October 2019 and February 2020. Eligibility criteria for the study included the following conditions: adults between 18-50 years of age who are not anemic (hemoglobin >10 g/dl), with normal medical history at screening and, for females, having a negative HCG test. All participants generally had a normal medical history at screening and physical examination. Up to 300 individuals, who met the eligibility criteria and provided written informed consent to be part of the study, were requested to donate venous blood samples. The *P. falciparum* infection status of participants was determined by microscopic examination of Giemsa-stained thick and thin blood smears, and later confirmed by PCR. Aside from collecting heparinized blood samples for cellular analysis (Kusi et al., 2022), 3 ml of venous blood was also collected from participants into Ethylenediaminetetraacetic Acid (EDTA) tubes for the preparation of plasma for serology. Whole blood and dried blood spots on Whatman No.3 filter paper were also prepared for molecular analysis.

### Amplification and sequencing of Pfama1 and Pfcsp

Genomic DNA was extracted from 250 μl of packed cells using the FlexiGene DNA kit (Qiagen, Hilden, Germany) following the manufacturer’s protocol. The *P. falciparum* infection status of each sample was determined using photo-induced electron transfer (PET) PCR targeting the *Pfr364* gene (Lucchi et al., 2013; Huang et al., 2020). PCR amplification was performed in a 20 µl reaction containing 2X TaqMan Environmental Master Mix 2.0 (Applied BioSystems, USA), 10 µM forward and reverse primers and 2 µl of DNA template. A no-template control was included as a negative control and DNA from a previously identified *P. falciparum* positive sample was included as a positive control. The standard curve was generated by performing 10-fold serial dilutions of a sample of the gDNA extracted from a *P. falciparum* 3D7 parasite culture with known concentration of 330 ng/μl. The reactions were subsequently loaded into a QuantStudio 3 real time PCR machine (Applied Biosystems, USA). The parasite density of all *P. falciparum*-positive samples was quantified using a six-step standard curve. The range of efficiencies generated for the runs were between 80 to 88%. The cycling conditions included an initial denaturation at 95°C for 15 minutes, followed by 45 cycles of denaturation at 95°C for 20 seconds, annealing at 63°C for 40 seconds and extension at 72°C for 30 seconds. A sample with a cycle threshold (CT) value below 40.0 was considered as positive. Samples that tested positive for *P. falciparum* by PET PCR were subsequently genotyped at the merozoite surface protein 2 locus using a protocol similar to that described by Adjah et al (Adjah et al., 2018).

The *Pfama1* and the *Pfcsp* genes were amplified from samples that yielded a single band after the *Pfmsp2* genotyping reaction (monoclonal for the msp2 gene). Amplification of *Pfama1* was performed in a 25 µl reaction containing 10X Accuprime buffer, 10 µM forward and reverse primers and 2U Accuprime Taq DNA polymerase. Two DNA samples from the 3D7 and Dd2 parasite strains that give different band sizes after amplification were used as positive controls in all reactions. Gene-specific primer-designs were based on the coding sequences of *Pfama1* of the *P. falciparum* 3D7 reference strain (PF3D7_1133400) downloaded from PlasmoDB version 50. Primers designed to amplify the different genes are listed in **Table 1**; *Pfama1* F1 and R3 were used for the initial reaction (1842 bp) and F1R2, F2R3 for the nested reactions (1185 bp and 1602 bp, respectively). The initial reactions were performed under the following cycling parameters: initial denaturation at 94°C for 2 minutes, followed by 35 cycles of denaturation at 94°C for 30 seconds, annealing at 50°C for 15 seconds and extension at 68°C for 2 minutes. For the nested reaction, the conditions were 35 cycles of denaturation at 94°C for 30 seconds and annealing was at 48°C for 15 seconds (F1R2) and 58.5°C for 15 seconds (F2R3).

**Table 1:** Primers used for the *Pfama*1 and *Pfcsp* reactions and the PET PCR.

Primer list	Primer sequences
*Pfama1*F1	ACTGCGTATTATTATTGAGCGCC
*Pfama1*R1	CATTGGTGACATAAGAGGTTCTG
*Pfama1*F2	AACCCGCACCACAAGAACA
*Pfama1*R2	CCCAATTATAACCCTTACCATGAC
*Pfama1*F3	TACATTGCTACTACTGCTTTGTCC
*Pfama1*R3	TTTCCATCAGAACTGGTGTTG
*Pfcsp*F1	GGCCTTATTCCAGGAATACCAGT
*Pfcsp*R1	GGATCTACATTTGGGTTGGCATTG
*Pfcsp*F2	ACCAGCGGATGGTAATCCTGA
*Pfcsp*R2	GGCATATTGTGACCTTGTCC
*Pfcsp*F3	ATCAAGGTAATGGACAAGG
*Pfcsp*R3	ACGACATTAAACACACTGGAAC
*P. falciparum* r364 For	ACCCCTCGCCTGGTGTTTTT
*P. falciparum* r364 Rev	AGGCGGATACCGCCTGGTCGGGCCCCAAAAATAGGAA (HEX-labelled)

Amplification of *Pfcsp* was performed in a 25 µl reaction containing 10X Accuprime buffer,10 µM forward and reverse primers, 50 mM MgSO_4_ and 2U Accuprime Taq DNA polymerase. Gene-specific primer designs were based on complete coding sequences of the 3D7 clone of *Pfcsp* (PF3D7_0304600) from PlasmoDB. Primers designed to amplify the gene are listed in **Table 1**; *Pfcsp* F1 and R3 were used for the primary reaction (1092 bp) and F1R2 and F2R3 were used for two independent nested reactions 807 bp and 955 bp, respectively).

The initial reactions were performed under the following cycling parameters: initial denaturation at 94°C for 2 minutes, followed by 35 cycles of denaturation at 94°C for 30 seconds, annealing at 57.1°C for 15 seconds and extension at 68°C for 1.15 minutes. Cycling parameters for the nested PCR comprised of 35 cycles of denaturation at 94°C for 30 seconds and an annealing step at 48.5°C for 15 seconds (F1R2) and 52.9°C for 15 seconds (F2R3). Amplified fragments of the *Pfama1* and the *Pfcsp* genes from 57 and 75 samples, respectively, were sent to Macrogen (UK) for commercial Sanger sequencing.

### Anti-PfAMA1 and anti-PfCSP antibody measurements

The levels of antibodies against recombinant PfAMA1 and PfCSP, as well as those against two conserved synthetic PfCSP peptides, were measured by in-house optimized ELISAs. The recombinant PfAMA1 antigen (amino acids 25 – 545) was based on the 3D7 parasite clone antigen (NCBI Reference Sequence: NC_037282), expressed in *Pichia pastoris* and purified by a methodology that has been previously described (Faber et al., 2008; Faber et al., 2016). The recombinant PfCSP antigen (amino acids 26 – 383) contained 4 NVDP and 38 NANP repeats (NCBI Reference Sequence: XM_001351086.1) from the 3D7 clone and was expressed in *Lactococcus lactis* as previously described (Singh et al., 2018; Singh et al., 2020). The PfCSP peptides were conserved 24mers from the NANPNVDP repeat region (NANPNVDPNANPNVDPNANPNVDP, amino acids 105 - 128) and the NANP repeat region (NANPNANPNANPNANPNANPNANP, amino acids 133 - 156), both based on the 3D7 clone PfCSP sequence and were commercially synthesized (JPT Peptide Technologies GmbH).

Recombinant antigens were coated at 1 μg/ml in a 96-well plate, while the synthetic PfCSP peptides were coated at 0.5 μg/ml for the measurement of plasma antibody levels. Briefly, antigen-coated, and blocked, ELISA plates (Maxisorp, NUNC, Denmark) were incubated with 100 μl/well of appropriately diluted test plasma samples in 1% non-fat milk in PBS. A pool of semi-immune sera, collected from adults with life-long exposure to malaria parasites, was used as a standard calibrator and titrated on each plate as an internal control. Plasma samples from study subjects were added to duplicate wells at 100 μl/well and incubated for two hours at room temperature. Afterwards, the plates were developed with 100 μl/well of 1:1000 dilution of goat anti-human IgG conjugated to horseradish peroxidase (Invitrogen, CA, USA) followed by incubation with the TMB Plus 2 substrate (KEM-EN-TEC, Taastrup, Denmark). After each reagent incubation, plates were washed four times with PBS containing 0.05% Tween 20 and the plates padded dry before the next reagent was added. The color reaction was stopped by the addition of 50 μl/well of 0.2M H_2_SO_4_ and optical densities (ODs) subsequently read at 450 nm using an automated 96-well ELISA plate reader (BioTek, VT, USA). To account for inter-plate and day-to-day variations, the measured OD data were converted into antibody titers using the 4-parameter logistic curve-fitting program ADAMSEL (Ed Remarque, available at: https://www.bprc.nl/sites/default/files/pubs/Adamsel-Suite.zip).

### Sequence and data analyses

DNA sequence data were base-called using the Chromas software (version 2.6.6). Base-called sequences were assembled using the BioEdit Sequence Alignment software and aligned using the online version of MAFFT multiple sequence alignment program (version 7). Aligned sequences were used to generate consensus sequences for each sample and realigned to the PlasmoDB 3D7 sequence for *Pfama1* (PF3D7_1133400) or *Pfcsp* (PF3D7_0304600).

Aligned sequences were translated to amino acids with the standard genetic code using MEGAX software. For PfAMA1, a total of 55/57 (96.5%) sequences for the region spanning amino acids 28 to 365 passed base calling and were translated and aligned with the PlasmoDB 3D7 reference strain amino acid sequence as well as the GenBank isolate sequences ETW15355.1 (isolate FV0), ACB87792.1 (isolate HB3), ACB87777.1 (isolate CAMP) and ABN12123.1. However, only fourteen out of the 55 samples were successfully sequenced for the second half of the protein spanning amino acid position 366 to 613 and assessed for genetic variation within this region.

A total of 69/75 (92%) *Pfcsp* sequences passed base-calling, although only 43 full sequences were obtained. All the 69 sequences were translated and aligned with the PlasmoDB 3D7 reference sequence as well as the GenBank sequence KOB59292.1 (isolate HB3).

Cladograms assessing the relatedness of the sequences were constructed in MEGAX software using the Maximum likelihood method based on the Jones Taylor Thornton model and bootstrapping at 500 replicates. Uniform rates among sites were allowed and initial trees for maximum likelihood were permitted using the neighbor joining and nearest neighbor interchange heuristic method. For each antigen, sequences were grouped based on their relatedness to any of the five reference sequences for comparison.

For each recombinant antigen and for the two synthetic PfCSP peptides, antibody levels were compared between PCR-positive and PCR negative individuals using the Mann-Whitney U test. For all antigens/peptides, sequence clusters from similar clades following phylogenic analyses were identified and used to create groups for antigen or peptide-specific antibody comparisons. Antibody levels were compared amongst the different clusters using the Mann-Whitney U test (recombinant PfCSP and synthetic peptides) or by the Kruskal-Wallis test (PfAMA1). The level of statistical significance was set at a p value of < 0.05.

## Results

### Participant information

A total of 300 participants were recruited for this study between October 2019 and February 2020. Subjects ranged in age from 18 to 50 years and included 66% males. The proportions of infected subjects as detected by blood smear/microscopy and Rapid Diagnostic Test (RDT) detection methods were comparable but a significantly higher proportion were parasite-positive by the more sensitive PCR method (p < 0.0001, Chi-Square test for proportions, **Table 2**). Infected persons’ parasite density ranged from 20 to 4,286 parasites per µl of blood by microscopy. A total of 171/300 (57%) study participants tested positive for *P. falciparum* by PET PCR and all infected persons were asymptomatic.

**Table 2:** Characteristics of study participants.

Demographic and clinical features	Outcome	value	P value
Gender	Male	66	
	Female	34	
Proportion RDT	Pos	16	< 0.0001
	Neg	84	
Proportion Microscopy	Pos	18	
	Neg	82	
Proportion PCR	Pos	64	
	Neg	36	

The proportions of parasite-positive and negative individuals using the three different methods were compared using a Chi-Square test for proportions.

### MSP2 genotyping

Typing of the *msp2* gene was performed to determine the diversity of circulating parasites within the study population. Genomic DNA from a total of 165/171 (96.5%) samples tested positive for either or both of the *Pfmsp2* allelic families, suggesting high quality of DNA. Upon agarose gel electrophoresis, *Pfmsp2* amplicon band sizes ranged from 150 bp to 600 bp for the IC3D7 family and from 220 bp to 500 bp for the FC27 family. Of the 165 participants with detectable infections, 51 were infected with parasites belonging to the IC3D7 family, 62 had parasites belonging to the FC27 family and 52 participants had multi-clonal infections and contained parasites belonging to both IC3D7 and FC27 families.

### Polymorphisms in pfama1 and pfcsp sequences

**Figure 1:**
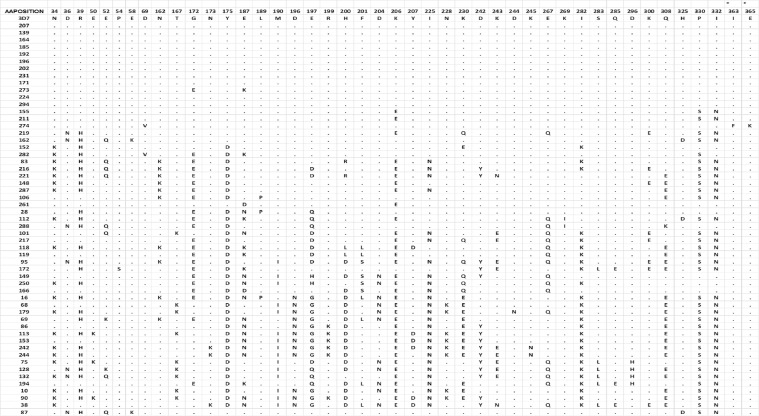
Amino acid variants based on reported and novel non-synonymous SNPs in PfAMA1 sequences. Data columns represent the polymorphic amino acid positions identified through sequencing, while rows represent the different clonal isolates whose sequences are being compared to the standard *P. falciparum* 3D7 antigen sequence. The symbol * at the top of some columns indicate the columns with novel amino acids that have been identified in this study.

Polymorphisms within the *Pfama1* and *Pfcsp* gene sequences of infecting parasites were identified by sequencing of the two genes using DNA from confirmed clonal infections. For the *Pfama1* gene, a total of 55 clonal parasite isolates were successfully analyzed and compared to the reference *P. falciparum* strain (3D7) PF3D7_1133400 for amino acid positions spanning amino acids 28 to 365. Eleven isolates had amino acid sequences identical to that of the 3D7 reference strain sequence. Five (9%) of the 55 variable sites were singletons, whilst 50/55 (91%) were parsimony informative. A total of 56 non-synonymous SNPs were identified for *pfama1* and resulted in 44 polymorphic positions including two novel positions (363 and 365, **Figure 1**) that have not been reported in the PlasmoDB SNP database. Five of the polymorphic positions were trimorphic (positions 52, 201, 230, 243 and 308), two were tetramorphic (positions 187 and 200) and a single site was pentamorphic (position 197).

**Figure 2:**
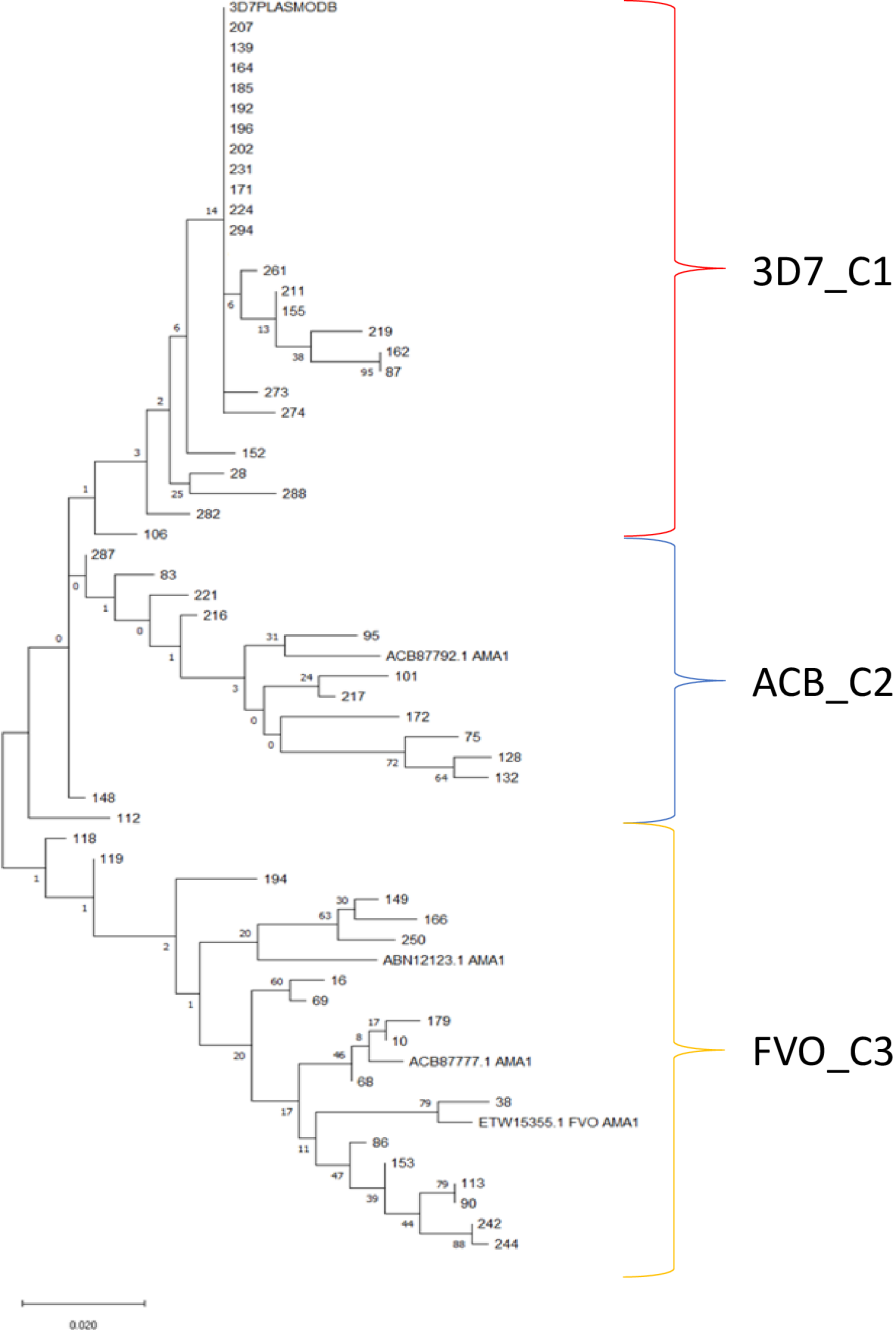
Maximum likelihood cladogram of 60 AMA1 amino acid sequences. These include 55 samples from our isolates and five reference sequences (3D7 consensus sequence, ETW15355.1, ACB87792.1, ACB87777.1 and ABN12123.1) from PlasmoDB or GenBank for comparison. The cladogram is drawn to scale with length of the branches measured in number of substitutions per site. 3D7_C1 represents the cluster of sequences with high level of relatedness to the 3D7 reference sequence, ACB_C2 is the cluster of sequences that are closely related to the ACB87792.1 (isolate HB3) reference sequence and FVO_C3 represents the cluster of sequences that are closely related to the ETW15355.1 (isolate FVO) reference sequence. Details of the reference sequences are presented in the Methods section.

The most frequently occurring amino acid change was P330S in 35 (64%) of the 55 sequenced samples, followed by I332N and K206E, each of which occurred in 34/55 (62%) of sequenced samples. Their corresponding reported rates in the PlasmoDB database (PlasmoDB :: JBrowse, n.d) were 92%, 74% and 66% respectively. The amino acid changes occurred primarily in domain I (amino acids 98 - 305) of the ectodomain of the *pfama1* gene (**Figure 1**). The PfAMA1 amino acid sequences clustered into 3 main clades with the 3D7 and ACB87792.1 in two different clades and the ETW15355.1, ACB87792.1 and ABN12123.1 sequences in the third clade (**Figure 2**). The aligned sequences for all PfAMA1 variants are presented as an additional file (S1 Appendix).

A total of 14 sequences were successfully amplified for the region spanning amino acids 366 to 613 and also compared to that of 3D7. A total of 18 variant sites were identified in this region compared to the 20 variants reported on PlasmoDB. One of the 14 samples had the same sequence as the 3D7 strain of the malaria parasite. The most frequent variation was the N439H, identified in 10/14 (71%) followed by the M451K identified in 9/14 (64%) then E405K in 8/14 (57%) of the samples. These mutations have been reported on the PlasmoDB website at frequencies of 52% for N439H, 49% for E405K and 57% (E405K), respectively (S2 Appendix). All but two of the variants identified here had already been reported in the PlasmoDB website. The 2 reported variants that were not identified here were the K395R (13%) and the E526V (2%) variants. Two of the 18 polymorphic sites (393 and 435) were trimorphic while the rest were dimorphic.

**Figure 3:**
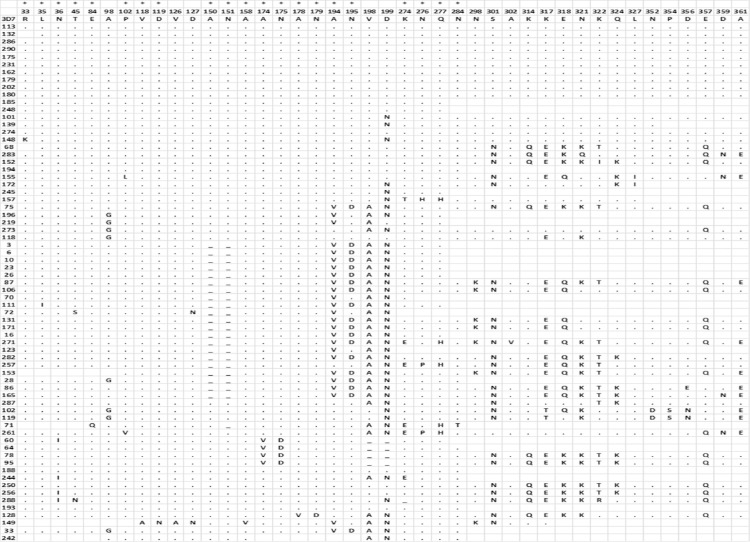
Amino acid variants based on reported and novel non-synonymous SNPs in PfCSP sequences. Data columns represent the polymorphic amino acid positions identified through sequencing, while rows represent the different clonal isolates whose sequences are being compared to the standard *P. falciparum* 3D7 antigen sequence. The symbol * at the top of some columns indicate the columns with novel amino acids that have been identified in this study.

For the *pfcsp* gene, a total of 69 clonal isolates were successfully analyzed for comparison. A total of 13/69 (18.8%) isolates had identical amino acid sequence as the 3D7 reference strain. There were 50 different non-synonymous SNPs that resulted in 42 polymorphic positions (**Figure 3**). Half (21) of the polymorphic positions were novel while the other half have been reported in the PlasmoDB database. Eight of the polymorphic positions were trimorphic (positions 45, 102, 274, 276, 317, 318, 321 and 356) while one was tetramorphic (position 322) (**Figure 3**).

**Figure 4:**
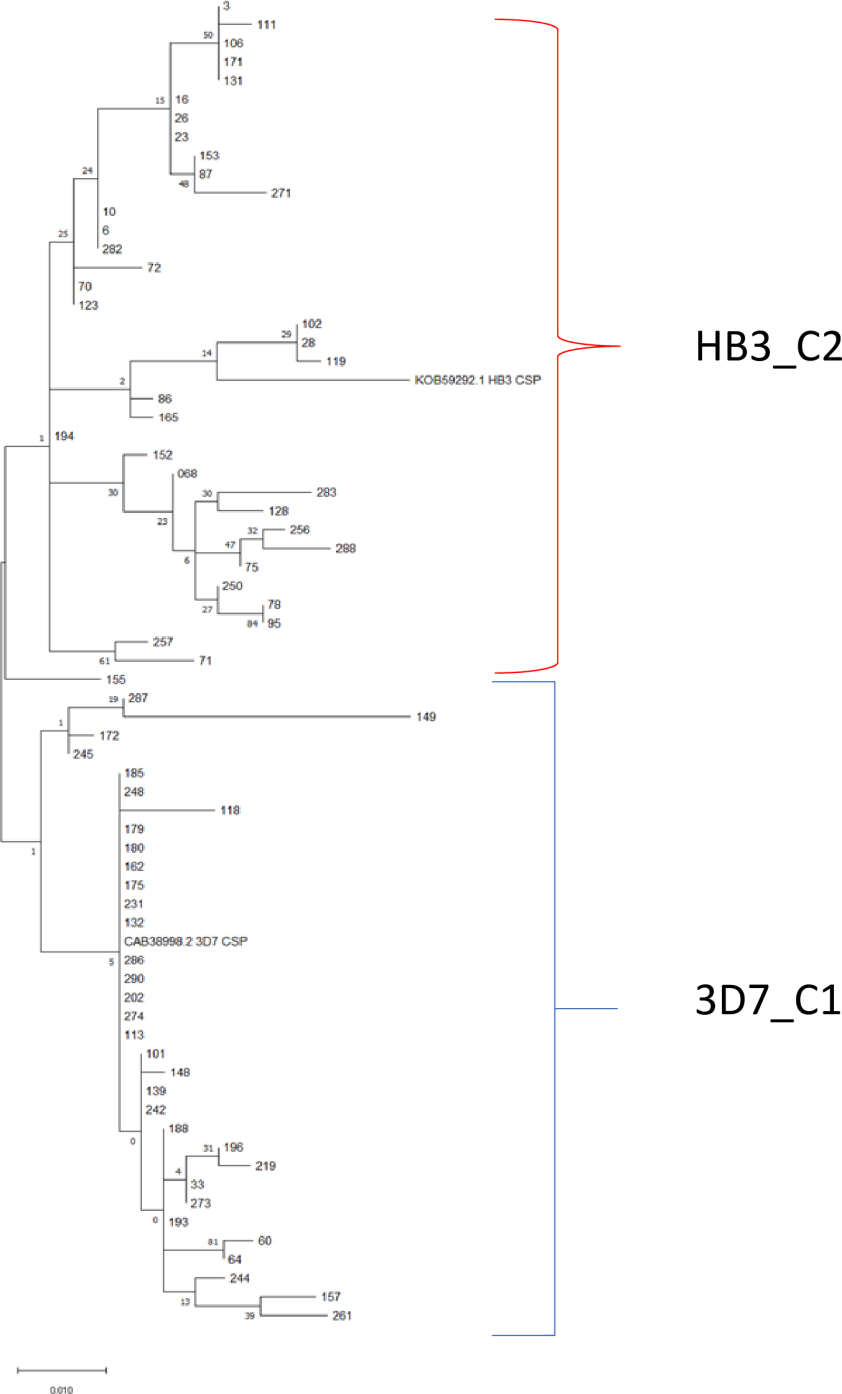
Maximum likelihood cladogram of 71 PfCSP amino acid sequences. These include 69 sequences from our isolates and two reference sequences from PlasmoDB for comparison. The cladogram is drawn to scale with length of the branches measured in number of substitutions per site. 3D7_C1 represents the cluster of sequences with high level of relatedness to the 3D7 reference sequence and the HB3_C2 is the cluster of sequences that are closely related to the KOB59292.1 (isolate HB3) reference sequence. Details of the reference sequences are presented in the Methods section.

The most frequently occurring SNPs resulted in amino acid changes V198A, D199N, S301N, K317E and E318K identified in 37/69 (54%), 45/69 (65%), 45/69 (59%), 24/44 (55%; 25 samples were incomplete) and 22/43 (51%; 26 samples were incomplete) of sequenced samples respectively. Their reported frequencies in the PlasmoDB database (PlasmoDB :: JBrowse, n.d) were 89%, 99%, 89%, 77% and 45%, respectively. Twelve of the 69 (17.4%) sequences contained an 18 amino acid peptide (DGNNNNGDNGREGKDEDKR) insertion (S3 Appendix) while 21 sequences had a deletion of the dipeptide ‘AN’ at amino acid positions 150 and 151 (**Figure 3**). It is noteworthy that all the novel mutations were identified between amino acid position 33 and 284. Phylogenetic analyses also identified two main clades after analyses of amino acid sequences, with the 3D7 and the KOB59292.1 (isolate HB3) sequences clustering in different clades (**Figure 4**).

### Antibody responses to recombinant antigens and synthetic peptides

**Figure 5:**
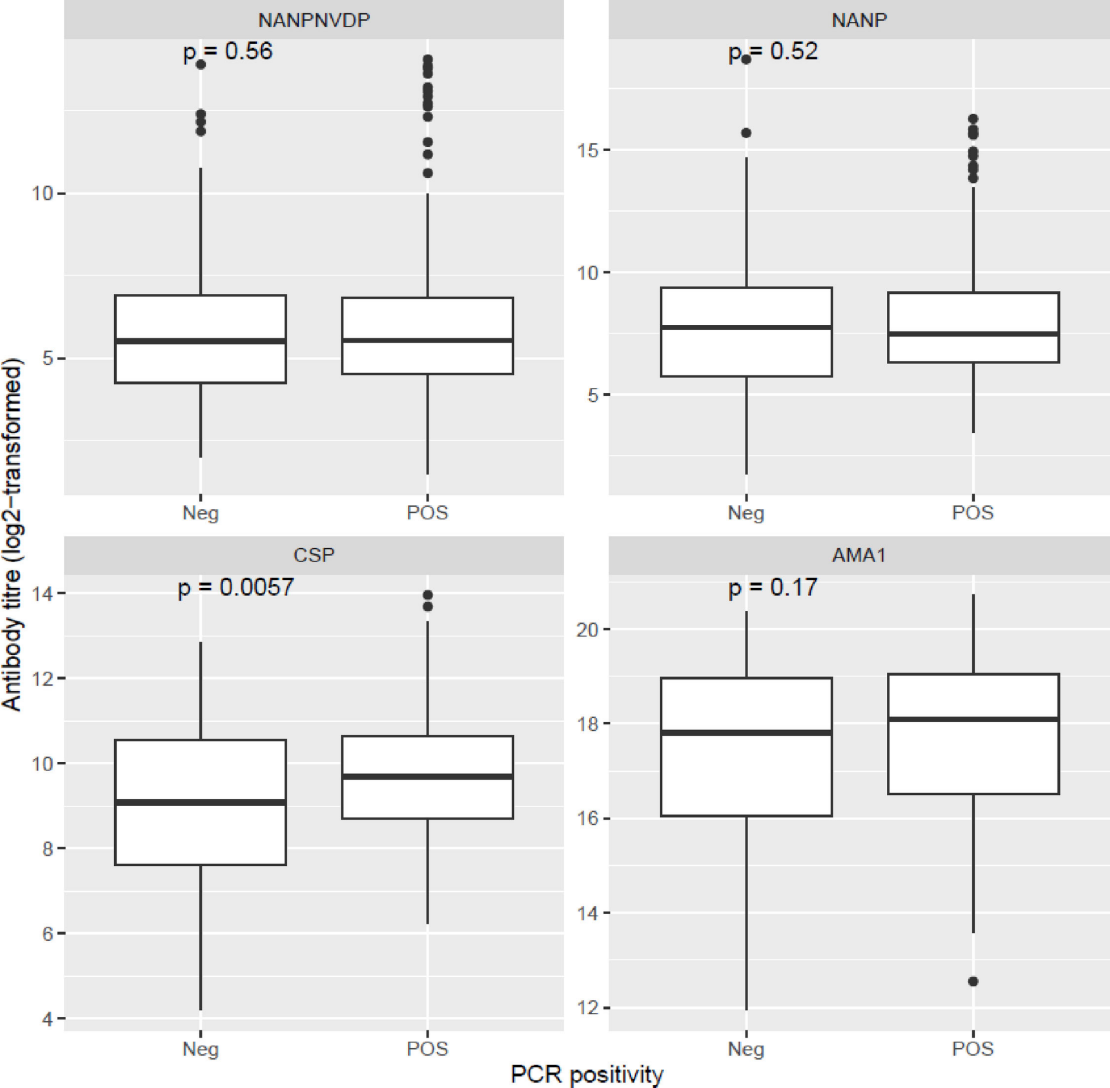
Levels of anti-PfAMA1 and anti-PfCSP antibody levels in the 300 study subjects. For each antigen/peptide, antibody levels were compared between PCR-positive and PCR-negative samples using box and whisker plots. The boxes represent the lower quartile, median and upper quartile of the data distribution and the whiskers are the 1.5 times the interquartile range. For each antigen/peptide, antibody titers were compared between parasite positive and parasite-negative subjects by the Mann-Whitney U test.

The levels of antibodies against the 3D7 strain recombinant PfAMA1 and PfCSP, as well as those against two synthetic 3D7 strain PfCSP peptides from the conserved middle region were assessed by an indirect ELISA. Antibody levels against PfAMA1, the 24mer NANP repeat peptide and the 24mer NVDPNANP repeat peptide were not significantly different between parasite-positive and parasite-negative individuals (**Figure 5**). Antibody levels against the recombinant PfCSP antigen was however significantly higher in individuals who were parasite-positive compared to those who were parasite-negative by PCR at the time of sampling (**Figure 5**).

### Effect of antigen diversity on antibody responses

**Figure 6:**
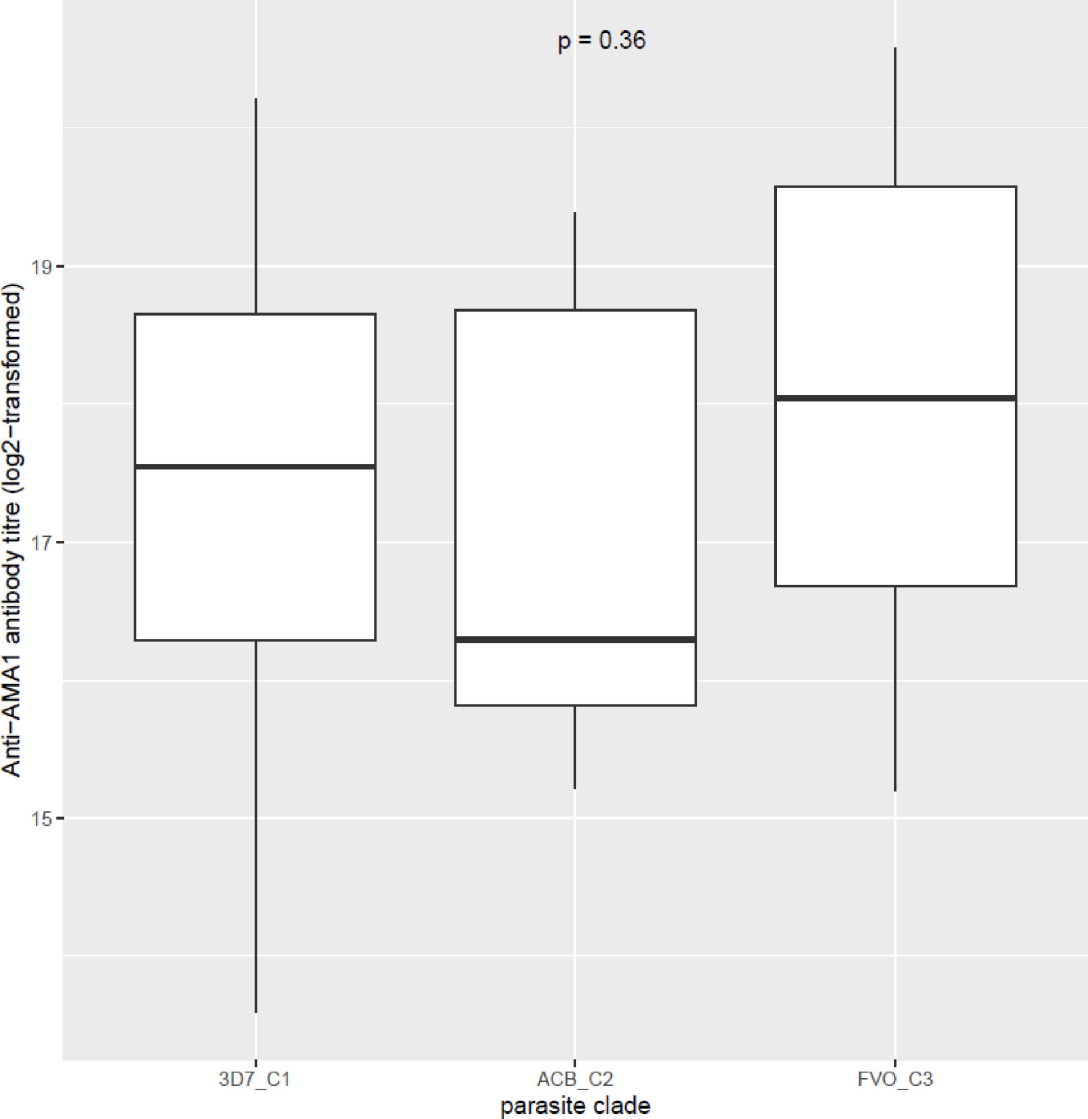
Levels of anti-PfAMA1 antibodies based on the different antigen sequence clusters. Antibody levels were compared amongst persons whose infecting parasites had antigen sequences phylogenetically determined to cluster with either the 3D7 parasite strain sequence (3D7_C1), the ACB_C2 strain sequence or with the FVO strain sequence (FVO_C3) using box and whisker plots. The boxes represent the lower quartile, median and upper quartile of the data distribution and the whiskers are the 1.5 times the interquartile range. Antibody titers were compared amongst the different parasite clades by the Kruskal-Wallis test.

**Figure 7:**
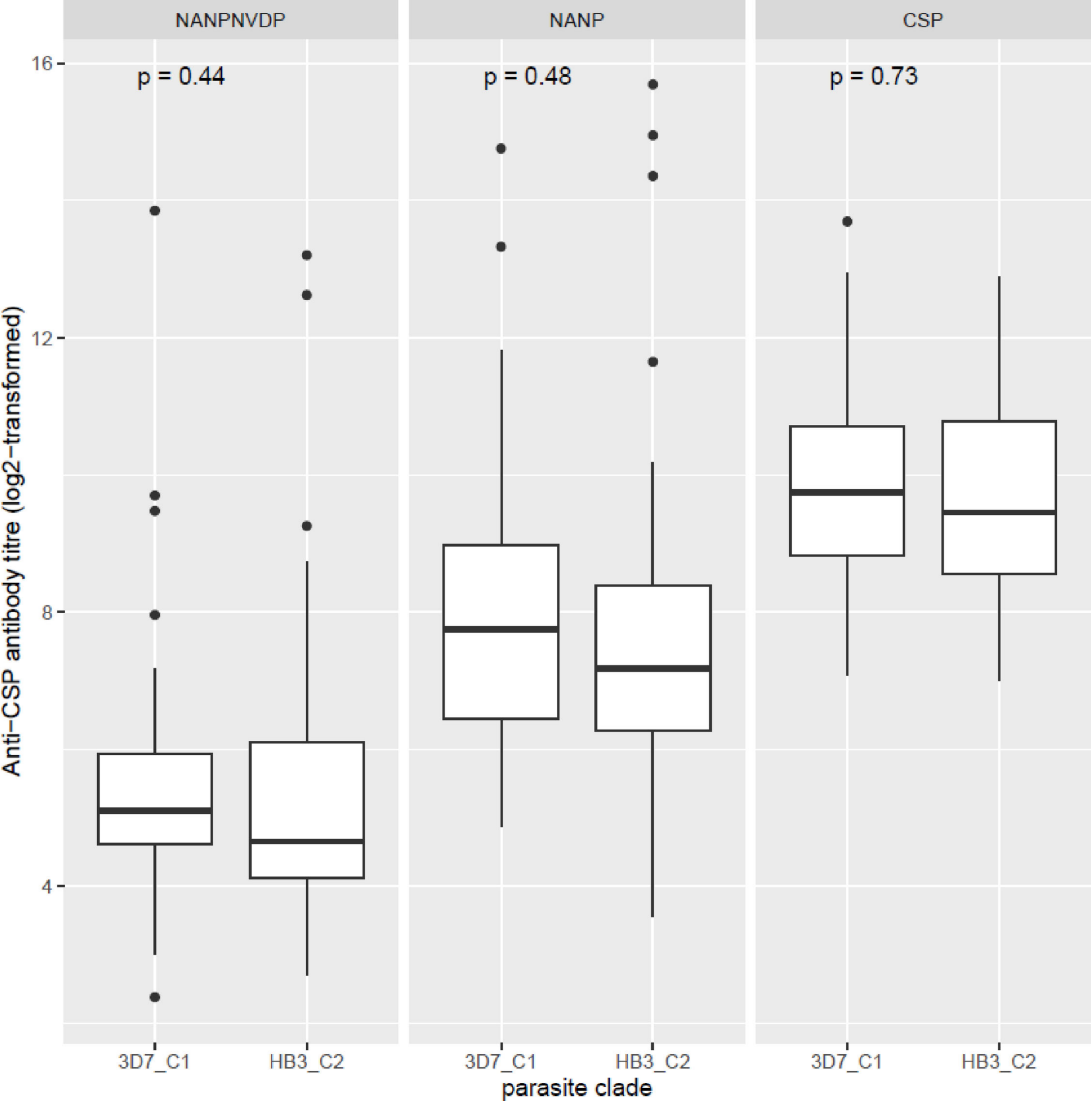
Levels of anti-PfCSP antibodies based on the different antigen sequence clusters. Antibody levels were compared between persons whose infecting parasites had antigen sequences phylogenetically determined to cluster with either the 3D7 parasite strain sequence (3D7_C1) or with the HB3 strain sequence (HB3_C2) using box and whisker plots. The boxes represent the lower quartile, median and upper quartile of the data distribution and the whiskers are the 1.5 times the interquartile range. Antigen or peptide-specific antibody titers were compared between the two parasite clades by the Mann-Whitney U test.

The effect of antigen diversity on antibody responses was assessed by comparing antibody levels between the gene cluster containing the 3D7 sequence, the antigen of which was used for antibody measurements, and the other identified clusters based on the phylogenic analyses. For the *pfama1* gene, three different clusters were identified (named 3D7_C1, ACB_C2 and FVO_C3) while for the *pfcsp* gene, two clusters were identified (named 3D7_C1 and HB3_C2). Antigen-specific antibody responses were compared amongst the three *pfama1* clusters (**Figure 6**) and between the two *pfcsp* gene clusters (**Figure 7**). For each of these comparisons, there were no statistically significant differences between the cluster with the 3D7 consensus sequence and the other cluster(s), suggesting a minimal effect of the identified polymorphisms on the elicited antibody responses.

## Discussion

Diversity in malaria parasite antigens is a significant and important barrier to the development of interventions such as vaccines and diagnostic tools, since antibodies raised against certain antigen targets may not be effective at recognizing other polymorphic variants of the same antigen. There is ample evidence of this effect on the induction of antibody responses (Remarque et al., 2012; Ouattara et al., 2013; Neafsey et al., 2015) and this may also extend to immune recognition of T cell epitopes within vaccine candidates (Nlinwe et al., 2021; Ofori et al., 2021). We therefore undertook an assessment of antigen diversity in the *P. falciparum* AMA1 and CSP antigens from circulating parasites in persons living in a moderate to high malaria transmission community of southern Ghana and further assessed the effect of these polymorphisms on the specificity of antibody responses in these persons.

Based on data from successfully analyzed sequences, this study confirmed the reported extensive diversity in both PfAMA1 and PfCSP (Escalante et al., 2002; Cortes et al., 2003; Farooq et al., 2009; Remarque et al., 2012). For PfAMA1, 44 polymorphic sites were identified, out of which 42 (95.5%) have been reported in the PlasmoDB SNP database and 2 (4.5%) of them are a result of novel SNPs. The location of these novel SNPs is interesting, as they are both located in the allegedly conserved “loop 2 domain” that stretches from amino acid 348 to 390. This loop has been functionally implicated in the invasion process of the RBC (Vulliez-Le Normand et al., 2012) and is the target of the strain-transcending inhibitory monoclonal antibody 4G2 (Pizarro et al., 2005). The extensive polymorphism in PfAMA1 has been widely reported (Escalante et al., 2001; Duan et al., 2008; Remarque et al., 2008; Remarque et al., 2012) and the identification of such a large number of polymorphic residues, including two novel polymorphic positions (363 and 365) is as expected. The SNPs R39H, Y175D, K206E, P330S and I332N were found in greater than 50% of the 55 isolates analyzed while SNPs E405K, N439H and M451K were also present in over 50% of the 14 isolates analyzed. Studies of East Asian parasites from Myanmar however found that the R39H, Y175D and P330S changes were fixed in all parasites while the N439H mutation occurred in over 70% of sequenced samples, as observed in this study. These loci may be indicative of regions of immune pressure and hence the need for their change to allow immune evasion (Bai et al., 2005).

Of the 42 polymorphic PfCSP positions identified, 21 were novel and resulted from 23 substitutions occurring in residue positions that have not been previously reported. All our identified novel SNPs however occurred at much lower frequencies, hence their true impact on immune escape to the resulting antigens may not be significant at the population level. However, the possibility of these variants being a source of immune-resistant clones upon selection cannot be ruled out. The SNPs A194V, N195D, V198A, D199N, S301N and K317E were identified in greater than 30% of the 69 isolates.

These collectively show the huge diversity in parasite variants that exist in our study population, and this is not surprising as it is a community with moderate to high malaria transmission. Areas with this level of transmission are believed to have a higher rate of recombination, and together with immune and/or drug pressure, these drive the generation of new parasite variants (Kyei-Baafour et al., 2020). The existence of these diverse variants in the same population will have significant implications for the success of vaccination programs since many candidates in development as well as the currently approved RTS,S/AS01 vaccine are all based on antigens from single alleles (Neafsey et al., 2015; Naung et al., 2022).

While only two novel polymorphic sites (4.5%) were identified for PfAMA1 in the study population, just about half of the polymorphic sites identified for PfCSP were novel with references to data from PlasmoDB. The higher frequency of novel epitopes in PfCSP compared to PfAMA1 suggests a possible pressure on the PfCSP antigen. The RTS,S/AS01 malaria vaccine contains a portion of the central repeat region and the C-terminal region of the PfCSP antigen. It has been extensively deployed in Ghana over the last 12 to 15 years, beginning with the phase 3 multi-country clinical trial of the candidate vaccine, which included two sites in northern Ghana (Asante et al., 2011; “Efficacy and safety of the RTS,S/AS01 malaria vaccine during 18 months after vaccination: a phase 3 randomized, controlled trial in children and young infants at 11 African sites,” 2014; Owusu-Agyei et al., 2009), to the WHO pilot roll-out of the vaccine in Ghana, Kenya and Malawi over the last four years (Praet et al., 2022; Adeshina et al., 2023; “WHO recommends groundbreaking malaria vaccine for children at risk,” n.d). Immune pressure may however not be a direct effect of vaccine deployment for two key reasons; first, the portion of PfCSP antigen that is in the vaccine covers amino acids 207 to 395 (Stoute et al., 1997), while most of the novel polymorphic positions found in this study are from amino acid positions 33 to 284. The overlap region between the two sequences (amino acid positions 207 to 284) therefore has just four of the novel sites, suggesting that vaccine immune pressure may not necessarily be driving the many other new mutations. Second, the study community (Obom in the Greater Accra Region) and its environs have not been one of the testing sites for the RTS,S vaccine over the years. Related to this, the current study population is from 18 to 50 years living in the south, while vaccinees within the RTS,S clinical trials/pilot testing have been children under 5 years, mostly within the middle and northern sectors of Ghana. Thus, the reason for the many new polymorphic sites in PfCSP antigen remains unclear. While these could be attributed to pressure on the PfCSP antigen from naturally acquired immunity (Pance, 2020), similar pressure would be expected to be on PfAMA1, but this was not the case since only two novel polymorphic positions were found for PfAMA1 in this study community. Although PfAMA1 has up to about 15% of the 622 amino being polymorphic, the overall structure of the antigen seems highly conserved (Chesne-Seck et al., 2005; Lim et al., 2014), suggesting that certain key residues responsible for maintaining the overall 3D structure of the protein are highly conserved and may not be easily mutated. Thus polymorphisms in PfAMA1 may be inherently restricted to certain residue positions, and the striking difference in the number of new polymorphic positions in PfAMA1 compared to PfCSP may be due to this PFAMA1 residue position restriction, which results in structural conservation.

Antibody levels were measured against the PfAMA1 and PfCSP recombinant antigens as well as against two conserved synthetic peptides from the central repeat region of PfCSP, and each of these was compared between study subjects who were parasite-positive and those who were parasite-negative by PCR at the time of sampling. Anti-PfCSP IgG levels differed significantly between these two categories of study subjects, and this is consistent with findings from our previous work in which we have evaluated anti-PfCSP antibody seroprevalence as a marker of recent exposure to parasites from infectious mosquito bites (Kusi et al., 2016; The RTS,S Clinical Trials Partnership, 2014). This utility of anti-PfCSP antibodies has also been demonstrated in other studies (Ramasamy et al., 1994; Campo et al., 2011). In contrast, antibody levels against the two conserved PfCSP peptides did not differ statistically between parasite-positive and parasite-negative individuals. Antibody levels against these synthetic peptides were far lower on a per mg basis compared to those against the PfCSP recombinant antigen, and this may have accounted for the lack of difference in the two groups. Antibody levels against PfAMA1 were also not significantly different between parasite-positive and parasite-negative individuals. PfAMA1 is a highly immunogenic antigen and anti-PfAMA1 antibodies are typically known to persist for much longer periods (Drakeley et al., 2005; Kusi et al., 2014). The levels of anti-PfAMA1 antibodies may therefore not be dependent on the current parasite infection status of individuals. Another possible reason for these general observations could be that our study population is exclusive made up of adults, who have been shown to develop a more cross-reactive immune response profile with age and exposure to different variants of the same antigen (Kusi et al., 2017). This observation, which we have described as epitope dilution (Kusi et al., 2018), is a result of the induction of clonal imprint responses through a focus of the immune response on epitopes that are shared between variant antigens of parasites that an individual has been exposed to over their life time, or the number of different parasites an individual is infected with at any time. We have also demonstrated this phenomenon with mixed and sequential variant antigen immunizations in rabbits (Kusi et al., 2011). Anti-malarial antibody responses may however be more strain-specific in children who have developing immune systems with fewer exposures to different parasite variants (Doolan et al., 2009; Tessema et al., 2019).

Some limitations of the current study include our inability to fully sequence the entire *pfama1* gene for many of the samples (41/55) as this reduces the power for discussing the diversity within the second half of the gene. This notwithstanding, the data reported here is very significant since there is very scant information on malaria parasite antigen polymorphism in any Ghanaian population.

In summary, the current study has corroborated the existence of huge diversity in the candidate malaria vaccine antigens PfCSP and PfAMA1, and, especially for PfCSP, identified a significant number of novel amino acid substitutions that have not been described elsewhere. These polymorphisms may however not have a significant impact on anti-malaria immune responses in adults but may rather be relevant in children, especially since the only approved malaria vaccine will be administered in children.

## Copyright statement

MS and EV are employees of the U.S. Government, and this work was prepared as part of their official duties. Title 17, U.S.C., §105 provides that copyright protection under this title is not available for any work of the U.S. Government. Title 17, U.S.C., §101 defines a U.S. Government work as a work prepared by a military service member or employee of the U.S. Government as part of that person’s official duties.

## Data availability statement

The original sequence data presented in the study are included in the article/**Supplementary Materials**. Also, sequence data have been deposited in the GenBank repository (accession numbers provided in separate worksheets in supplementary files 1 and 3). Further inquiries can be directed to the corresponding author.

## Ethics statement

The studies involving humans were approved by Noguchi Memorial Institute for Medical Research Institutional Review Board (NMIMR-IRB) and the Naval Medical Research Center Institutional Review Board (NMRC-IRB). The studies were conducted in accordance with the local legislation and institutional requirements. The participants provided their written informed consent to participate in this study.

## Author contributions

KK: Validation, Conceptualization, Data curation, Formal analysis, Funding acquisition, Investigation, Project administration, Resources, Supervision, Writing – original draft, Writing – review & editing. LA: Conceptualization, Formal analysis, Investigation, Methodology, Supervision, Writing – original draft, Writing – review & editing. FA: Data curation, Formal analysis, Methodology, Writing – original draft. NE: Data curation, Formal analysis, Methodology, Writing – original draft. AFF: Data curation, Formal analysis, Methodology, Writing – review & editing. EO: Data curation, Formal analysis, Investigation, Methodology, Writing – review & editing. KA-M: Data curation, Formal analysis, Investigation, Methodology, Writing – review & editing. EK-B: Data curation, Formal analysis, Investigation, Methodology, Validation, Writing – review & editing. FO: Data curation, Investigation, Methodology, Writing – review & editing. AF: Data curation, Investigation, Methodology, Supervision, Writing – review & editing. SS: Investigation, Methodology, Resources, Writing – review & editing. MT: Resources, Writing – review & editing, Data curation, Investigation. ER: Investigation, Methodology, Resources, Software, Writing – review & editing. BF: Investigation, Methodology, Resources, Software, Writing – review & editing. MB: Formal analysis, Investigation, Methodology, Resources, Writing – review & editing. HG: Data curation, Methodology, Resources, Writing – review & editing. JH: Investigation, Methodology, Resources, Writing – review & editing. EV: Conceptualization, Funding acquisition, Resources, Validation, Writing – review & editing. MS: Conceptualization, Funding acquisition, Investigation, Project administration, Resources, Validation, Writing – review & editing.
