# Supplementary figures and images for: Plasmodium falciparum AMA1 and CSP antigen diversity in parasite isolates from southern Ghana
Source: Front Cell Infect Microbiol. 2024 May 13;14:1375249. doi: 10.3389/fcimb.2024.1375249 (PMC11132687; doi:10.3389/fcimb.2024.1375249)

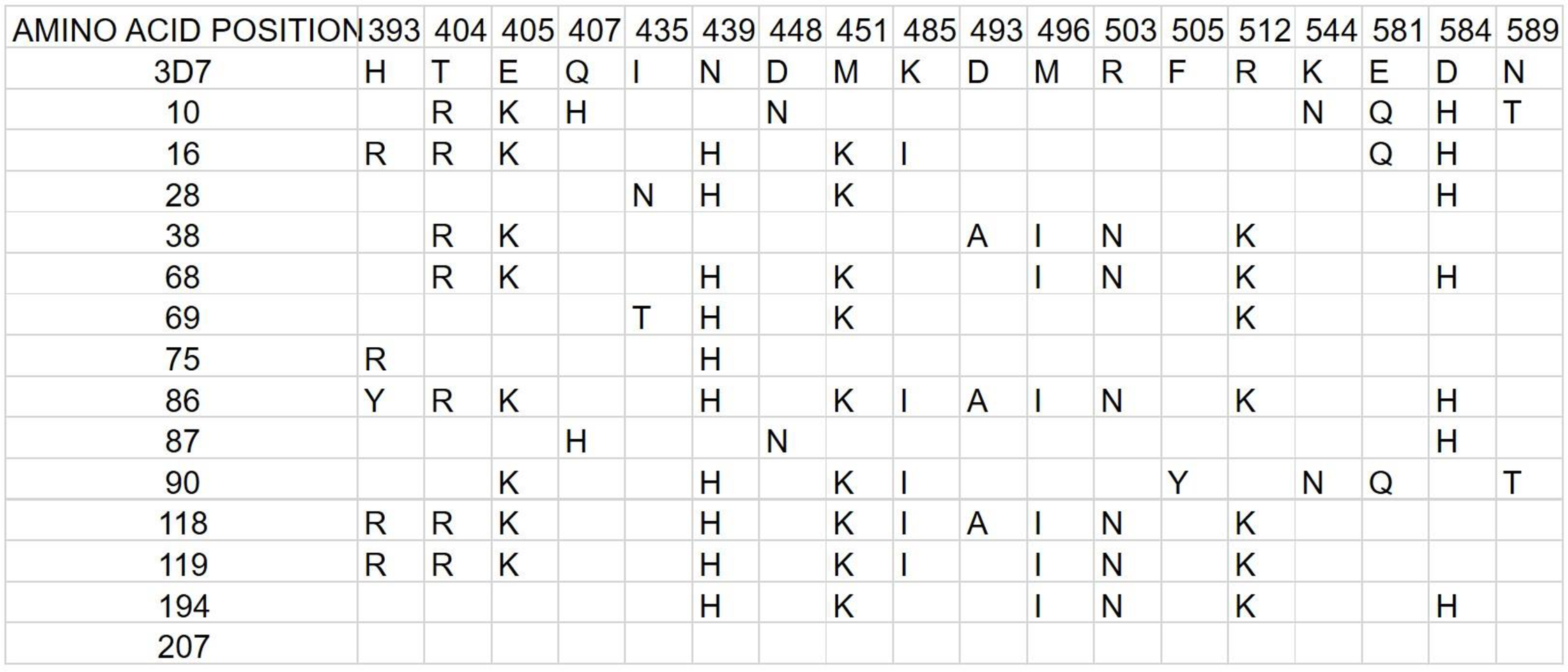

Supplement: Supplementary file 1 [file Image_1.tif]
